# Pancreatic adenocarcinoma with distant soft tissue metastases: a case report and literature review

**DOI:** 10.3389/fsurg.2025.1447865

**Published:** 2025-05-09

**Authors:** Xiangying Wang, Yang ChangSheng, Liu Yun, Geng Xiaodan

**Affiliations:** ^1^ Department of Orthopedic Oncology Surgery, Shandong Cancer Hospital and Institute Affiliated to Shandong First Medical University, Jinan, Shandong, China; ^2^ Department of Gastrointestinal Surgery, Shandong Provincial Hospital Affiliated to Shandong First Medical University, Jinan, Shandong, China; ^3^ Department of Radiology, Shandong Cancer Hospital and Institute Affiliated to Shandong First Medical University and Shandong Academy of Medical Science, Jinan, Shandong, China

**Keywords:** soft tissue, metastasis, distant, pancreatic cancer, cutaneous metastases, Sister Mary Joseph's nodes

## Abstract

**Background:**

Pancreatic cancer is a common malignant tumor that is highly aggressive and can develop distant metastases at an early stage. Pancreatic cancer metastasis often occurs in localized lymph nodes, peritoneum, lungs, and liver. The occurrence of distant soft tissue metastases, especially of skeletal muscles and subcutaneous tissues, is extremely rare. Here, we report a case of pancreatic adenocarcinoma metastatic to the skin of the shoulder.

**Case report:**

We have reported here the case of a middle-aged female patient who was admitted to the hospital with a tumor in her right shoulder and a complaint of severe pain for the past 1 month. After completing the relevant examinations, her tumor was entirely surgically resected, and the postoperative pathological result suggested that the pancreatic adenocarcinoma had metastasized. Next, a computed tomography (CT) scan of the chest, abdomen, and pelvis revealed multiple metastases of pancreatic head cancer. Presently, the patient is at an advanced stage of pancreatic cancer and cannot undergo radical surgery. Accordingly, the patient has been referred to the Department of Gastroenterology for comprehensive treatment. We have discussed this rare case in the light of the literature.

**Conclusion:**

Distant soft tissue metastasis of pancreatic cancer is extremely rare and is often detected at an advanced stage, thereby showing a poor prognosis. As this cancer can metastasize very fast and is difficult to resect, specific treatment measures must be taken promptly based on the patient's history, symptoms, and clinical manifestations for a comprehensive treatment.

## Background

Pancreatic cancer is a highly aggressive malignant tumor with a 5-year survival rate of about 10% (1, 2). Since the pancreas is located in the retroperitoneum, the disease is often in a locally progressive or advanced stage when clinical symptoms appear. When pancreatic cancer metastasizes, it usually metastasizes to the liver, retroperitoneum, lungs, and bones (3). Cutaneous metastases are very rare. The most common primary tumors with cutaneous metastases are lung, kidney, and colon cancers (4), and cutaneous metastases from pancreatic cancer are very rare. When cutaneous metastases occur, they are usually seen in the navel and are called Sister Mary Joseph's nodes(SMJN) (5). Non-umbilical cutaneous metastases are even rarer. This study reports a case of pancreatic adenocarcinoma metastatic to the cutaneous part of the shoulder.

## Case report

**Figure 1:**
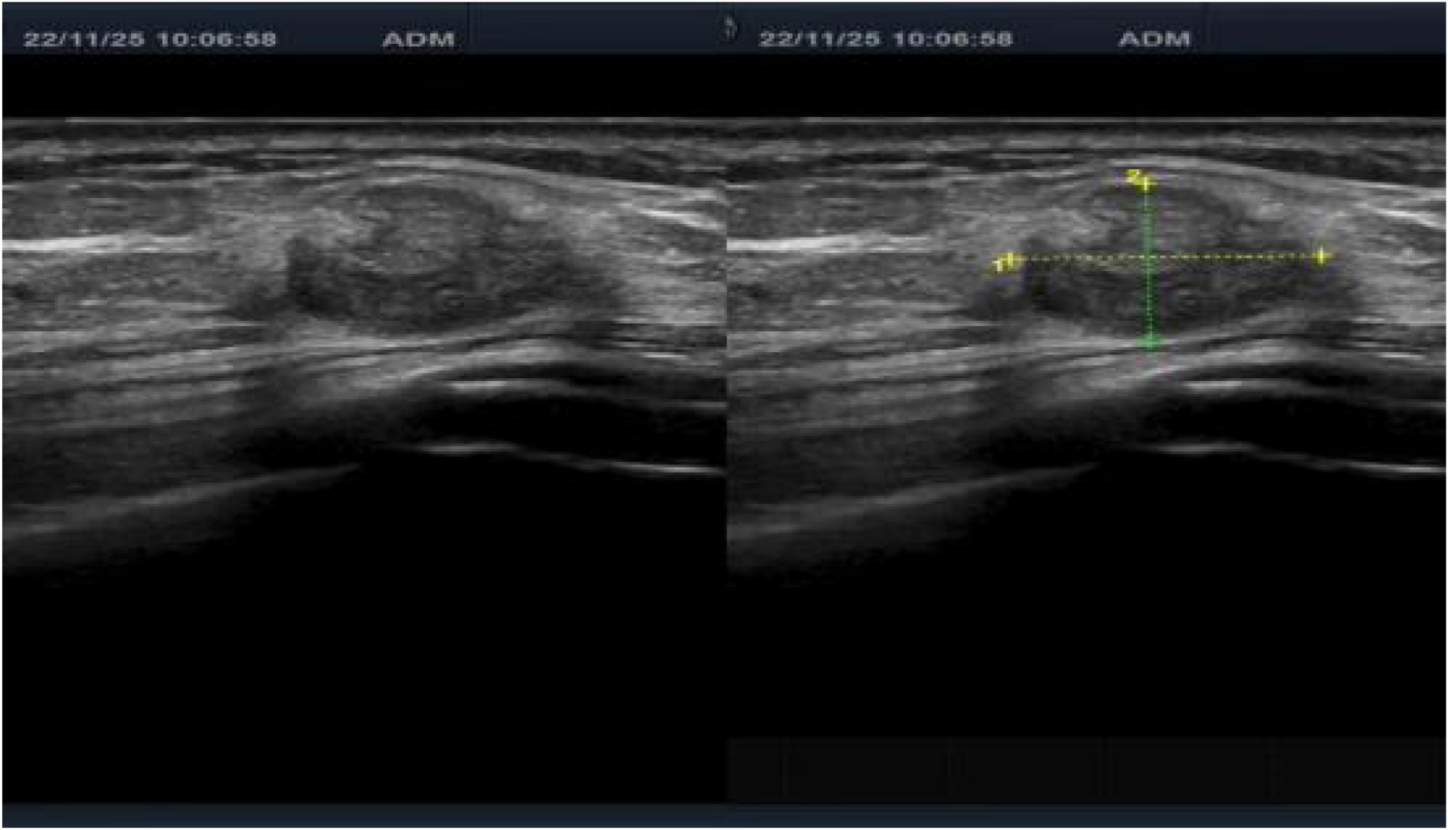
Preoperative ultrasound finding showing the tumor mass in the right shoulder.

**Figure 2:**
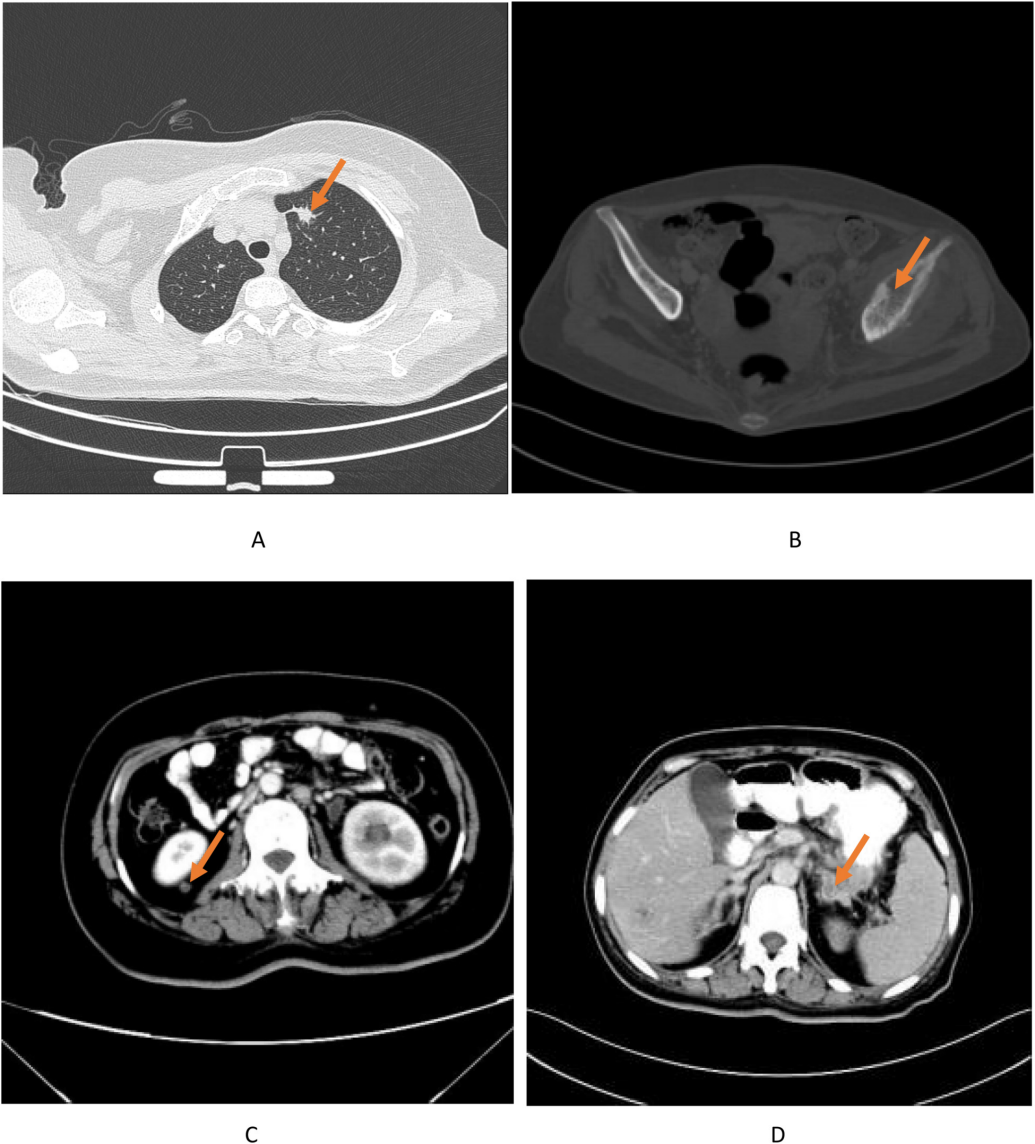
Post-operative CT scan findings showing multiple metastases of pancreatic head. Cancer to the **(A)** left lung **(B)** left iliac bone, **(C)** right kidney **(D)** left adrenal gland.

**Figure 3:**
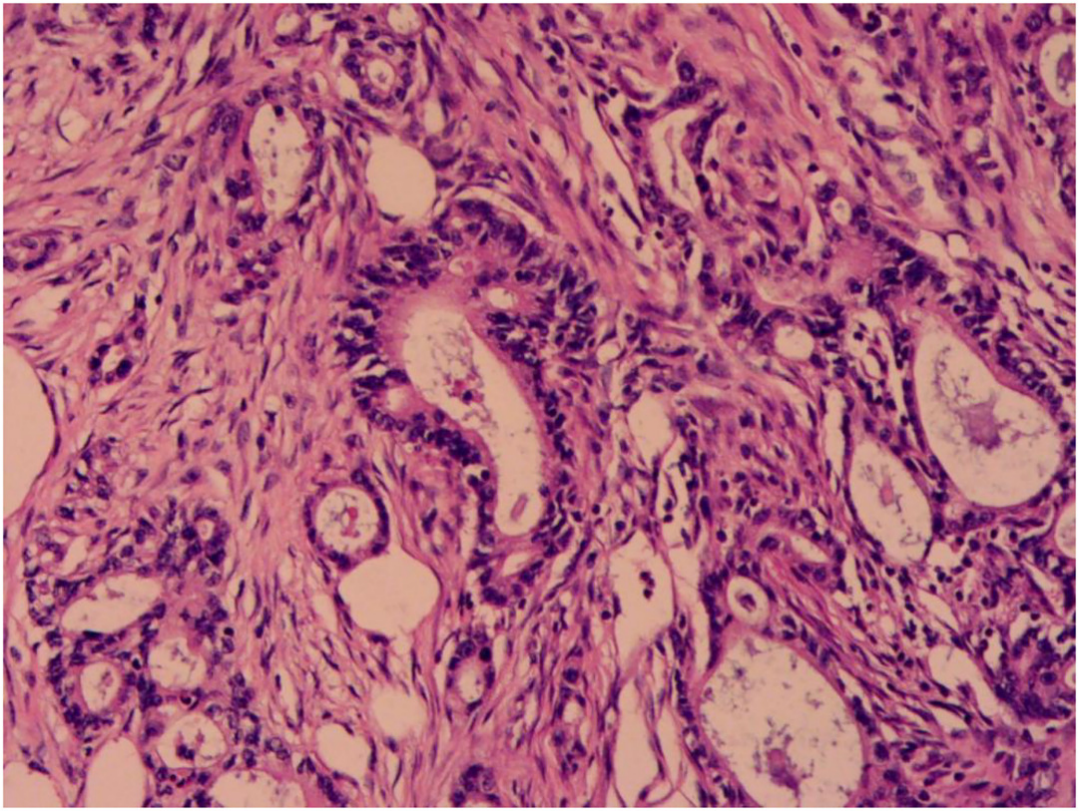
Pathology shows adenocarcinoma of the pancreas.

**Figure 4:**
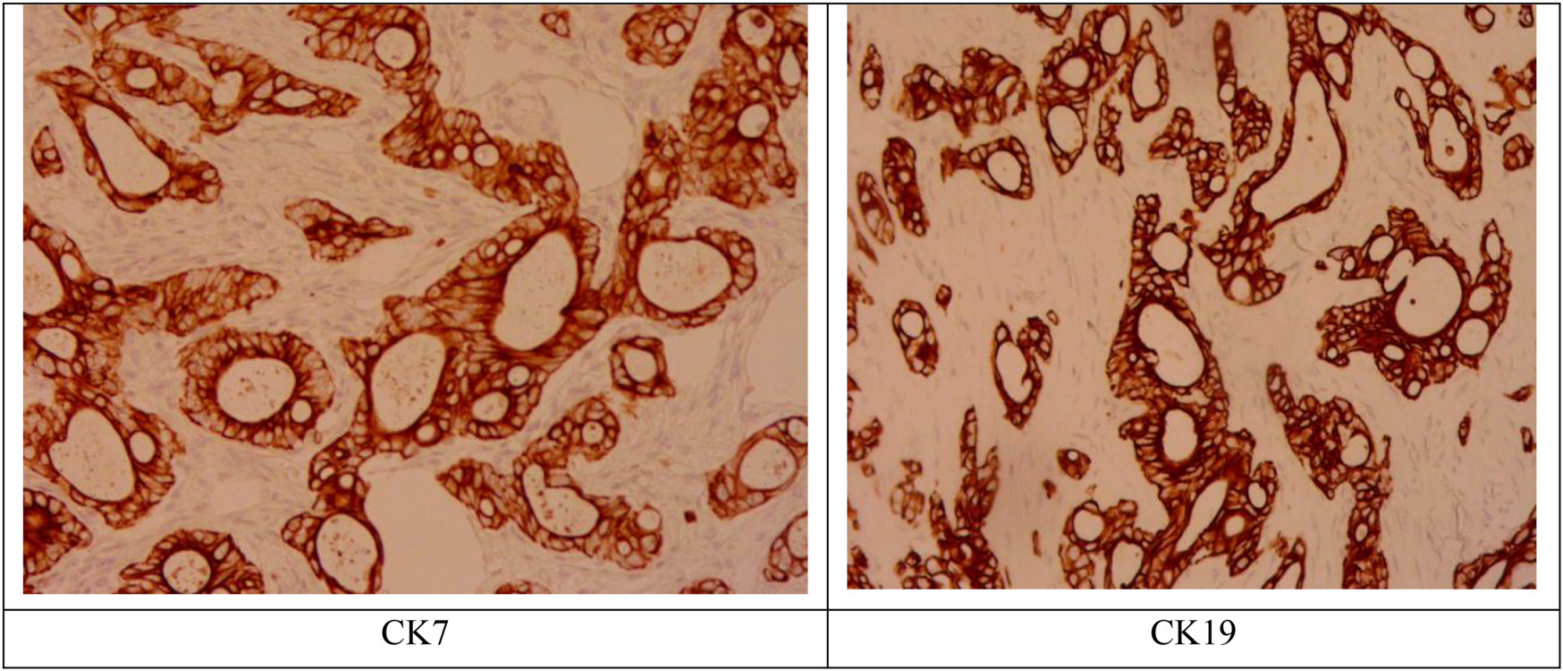
Immunohistochemistry results positive for CK7, CK19.

A 50-year-old woman was admitted to the hospital with “a tumor in the right shoulder with severe pain for the past 1 month”. On examination after admission, a tumor was detected in her right shoulder. The mass was round (approximately 3 cm diameter) with slightly reddish skin on the surface, a slightly high skin temperature, and average mobility. After admission, ultrasound revealed a 2.8 × 2.2 cm hypoechoic mass in the soft tissue of the right shoulder, with fuzzy boundary, heterogeneous echogenicity, and a small amount of blood flow signal detected in the tumor, which excluded the possibility of a metastatic tumor (Figure 1). Considering that the patient's right shoulder tumor was <4 cm in size, complete resection was deemed feasible. Accordingly, enhanced MRI or puncture biopsy was not performed to further clarify the diagnosis, and a wide complete resection of the tumor and pathological examination was undertaken. Subsequently, the patient underwent a whole-body CT examination, which indicated that the pancreatic head cancer was combined with multiple metastases in the liver, left adrenal gland, right kidney, bilateral uterine adnexes, retroperitoneal lymph nodes, chest wall, and pelvis (Figure 2). Postoperative pathologic examination suggested metastasis of pancreatic adenocarcinoma, and immunohistochemistry showed positivity for CK7, CK19, and CDX2 (Figures 3, 4). Presently, the patient is at an advanced stage of pancreatic cancer and cannot undergo radical surgery. Accordingly, we have transferred the patient to the Department of Gastroenterology for further comprehensive treatment.

Meanwhile this study conducted a comprehensive literature search in three major databases, viz.MEDLINE/PubMed, EMBASE and SCOPUS without any language restriction. The search strategy was performed using the following search string: “Pancreatic cancer skin metastasis”; “Pancreatic cancer skin metastasis OR Pancreatic cancer skin metastasis OR Pancreatic tumor skin metastasis OR Pancreatic tumor skin metastasis OR Pancreatic tumor skin metastasis”. A total of 60 cases were collected. The relevant information is summarized in Tables 1, 2 as follows.

**Table 1:** Cutaneous metastases characteristics in *n* = 60 patients.

Location	Count	Percentage
Umbilicus	14	23.3%
Other sites	44	73.3%
Head and neck	20	33.3%
Trunk	12	20%
Multiple sites	8	13.3%
Lower limbs	4	6.7%
Upper limbs	2	3.3%
Genital skin	1	5%
Clinical appearance		
Nodule/Mass/Plaque	53	88.3%
Swelling	4	6.7%
Ulceration	3	5%
Associated symptoms		
No symptoms	22	36.7%
Pain	16	26.7%
Bleeding	10	16.7%
Dimension		
>1 cm	41	68.3%
<1 cm	12	20%
Number		
Isolated metastases	40	66.7%
Multiple metastases	20	33.3%
Diagnosis		
Before pancreatic cancer	36	60%
After pancreatic cancer	23	38.3%
Concomitant to pancreatic cancer	1	1.7%

**Table 2:** Pancreatic cancer characteristics in *n* = 60 patients with cutaneous metastases.

Primary site	Count	Percentage
Head	25	41.6%
Body and tail	27	45%
Dimension		
<4 cm	31	51.7%
>4 cm	29	48.3%
Histology of skin metastases		
Adenocarcinoma	43	71.7%
Neuroendocrine cancer	4	6.7%
Sarcoma	2	3.4%
PDAC	1	1.7%
Signs and symptoms		
Gastrointestinal symptoms	24	40%
Jaundice	11	18.3%
Weight loss	11	18.3%
Lymph node metastases		
No	29	48.3%
Yes	21	35%
Distant metastases		
No	15	25%
Yes	39	65%
Liver	22	36.7%
Lungs	13	21.7%
Peritoneum	7	11.7%
Bones	5	8.3%
Portal vein	2	3.3%

## Discussion

Although the soft tissues of the human body account for about half of the body weight, the presence of soft tissue metastases in distant metastases of malignant tumors remains a very rare occurrence, accounting for approximately 0.36% of all soft tissue tumor cases (6). Soft tissue metastases may involve the skin, subcutaneous tissues, or muscle and may present as isolated lesions or as a disseminated disease. Generally, patients present to the clinic for the first time with distant soft tissue metastases from malignant tumors, as in the present case, which may seem to be diseases with an unknown primary focus. At this point in time, when distant soft tissue metastases have occurred, the disease is often at an advanced stage and has a poor prognosis. According to the literature, the average survival time for malignant tumors with distant soft tissue metastases is 1–19 months (7). In a study of primary diseases with distant soft tissue metastasis, the most frequently reported cases of malignant tumors with distant soft tissue metastasis are of lung cancer, followed by renal cancer, colon cancer, unknown diseases, and pancreatic cancer (in 1 case) (4, 8), suggesting that pancreatic cancer with distant soft tissue metastasis was very rare. When cutaneous metastases occur, they are usually seen in the navel and are called Sister Mary Joseph's nodes (SMJN). Metastases to other parts of the skin are very rare.

The diagnosis of soft tissue metastasis of malignant tumors is challenging, and the differential diagnosis mainly depends on the anatomical location of the disease and the tissues invaded by the tumor (e.g., skin, subcutaneous tissues, and skeletal muscles), including skin diseases, primary soft tissue sarcomas, and some non-tumor diseases like abscesses and ossifying myositis (9). The main clinical symptom of malignant tumor soft tissue metastasis is pain, especially when the tumor invades muscle tissues (10). Currently, the main diagnosis is based on the patient's medical history, imaging, and puncture biopsy. Fine-needle aspiration biopsy is the main diagnostic method, and pathological diagnosis is the gold standard. Considering that the patient's tumor was small in size and could be completely resected, we did not perform a puncture biopsy on the patient, but rather a complete excisional biopsy. Postoperative pathologic examination suggested pancreatic adenocarcinoma. Immunohistochemistry result CK7, CK19 positive confirmed the above result.

The treatment of soft tissue metastases from malignant tumors depends largely on the site of the primary disease, the patient's symptoms, the general condition, and the type and extent of the metastatic tissues. Past studies have revealed that the presence of distant metastases from primary cancer often represents a poor prognosis, with an overall average survival of 15.36 months, with lung cancer showing the worst prognosis at 6.7 months (11). Median survival for patients with cutaneous metastases from pancreatic cancer is only 5 months (12).Therefore, the treatment of malignant tumors after the development of distant metastases is generally palliative, which includes monitoring, radiotherapy, chemotherapy, or resection. Although the excision of small lesions for diagnostic purposes (excisional biopsy) or to control excessive pain is generally acceptable, wide excision for therapeutic purposes should be performed only in highly selected cases. Patients in good general condition, with isolated soft tissue metastases, successful treatment of primary disease, no progression of the disease over a longer period, and good histology may be considered for surgical resection treatment. For patients with scalp skin metastases of pancreatic cancer with ulcers, the main treatment for pancreatic cancer is usually gemcitabine combined with paclitaxel chemotherapy (3). For the detection of multiple metastases of pancreatic cancer, PET-CT is the first choice, the patient's family refuses to undergo PET-CT due to the patient's financial reasons. So we further performed enhanced CT of the chest abdomen and pelvis in this patient, which revealed that pancreatic cancer with multiple intrahepatic metastases, abdominal and retroperitoneal lymph node metastases, left adrenal metastases, right kidney metastases, chest wall metastases, bilateral adnexal area metastases, right inguinal metastases, and multiple bone metastases. The most sensitive tumor marker for pancreatic adenocarcinoma is CA19-9. After admission, the results of CA19-9 examination showed that it was more than 10,000 U/ml. Considering the advanced stage of pancreatic cancer, there was no scope for radical surgical resection, and the patient was transferred to the Department of Gastroenterology for further comprehensive treatment. Paclitaxel and gemcitabine chemotherapy was given in the gastroenterology department, and CA19-9 was periodically reviewed during the treatment, and the values were all greater than 10,000 U/ml; the patient died 4 months after the diagnosis of pancreatic cancer.

## Conclusion

Soft tissue metastases from primary malignancies are extremely rare, with the usual primary site being the lungs, kidneys, and colon. Distant soft tissue metastases from pancreatic cancer are even rarer. Although the presence of distant soft tissue metastases from pancreatic cancer indicates advanced disease, with a median survival of only about 5 months. Complete resection of the lesion for diagnosis, pain relief palliation seems to be a feasible option for small, isolated, soft tissue metastases only.

## Data Availability

The datasets presented in this study can be found in online repositories. The names of the repository/repositories and accession number(s) can be found below: This is a case report and does not need to meet specific ethical requirements.
